# Characterising vincristine-induced peripheral neuropathy in adults: symptom development and long-term persistent outcomes

**DOI:** 10.1007/s00520-024-08484-5

**Published:** 2024-04-09

**Authors:** Tiffany Li, Terry Trinh, Annmarie Bosco, Matthew C. Kiernan, David Goldstein, Susanna B. Park

**Affiliations:** 1 Brain and Mind Centre, The University of Sydney, Sydney, Australia; 2 School of Medical Sciences, Faculty of Medicine and Health, The University of Sydney, 94 Mallett St Camperdown, Sydney, NSW 2050 Australia; 3 Prince of Wales Clinical School, University of New South Wales, Kensington, Australia; 4 Prince of Wales Hospital, Randwick, Australia; 5 Department of Neurology, Royal Prince Alfred Hospital, Sydney, Australia

**Keywords:** Vincristine-induced peripheral neuropathy, Natural history, Phenotype, Patient reported outcomes, Nerve conduction

## Abstract

**Background:**

Decades following the introduction of vincristine as treatment for haematological malignancies, vincristine-induced peripheral neuropathy (VIPN) remains a pervasive, untreatable side-effect. However there remains a gap in understanding the characteristics of VIPN in adults. This study presents a comprehensive phenotyping of VIPN.

**Methods:**

Adult patients (*n* = 57; age = 59.8 ± 14.6) were assessed cross-sectionally following completion of vincristine (months post treatment = 16.3 ± 15.6, cumulative dose = 7.6 ± 4.4), with a subset of 20 patients assessed prospectively during treatment. Patient reported measures (EORTC-QLQ-CIPN20, R-ODS) were used to profile symptoms and disability. Neurological assessment was undertaken using the Total Neuropathy Score and nerve conduction studies. Sensory threshold and fine motor tasks were also undertaken. Comparisons of data between timepoints were calculated using paired-sample *t* tests or Wilcoxon matched-pairs signed-rank test. Comparisons between outcome measures were calculated with independent sample *t* tests or Mann–Whitney *U* tests for non-parametric data.

**Results:**

The majority of patients developed VIPN by mid-treatment (77.8%, 7.0 ± 3.3 weeks post baseline) with the prevalence remaining stable by end-of-treatment (75%, 8.1 ± 1.7 weeks post mid-treatment). By 3 months post-completion, 50% of patients still reported VIPN although there were significant improvements on neurological grading and functional assessment (*P* < 0.05). VIPN presented with sensorimotor involvement in upper and lower limbs and was associated with decreased sensory and motor nerve amplitudes, reduced fine-motor function and increased disability.

**Conclusion:**

VIPN in adults presents as a sensorimotor, upper- and lower-limb neuropathy that significantly impacts disability and function. Neuropathy recovery occurs in a proportion of patients; however, VIPN symptoms may persist and continue to affect long-term quality of life.

**Supplementary Information:**

The online version contains supplementary material available at 10.1007/s00520-024-08484-5.

## Introduction

Vincristine is an anti-cancer agent commonly used to treat haematological malignancies including lymphoma and leukaemia in both adults and children. Advancements in treatment protocols have resulted in sustained improvement in survival rates for patients with haematological malignancies [1]. Consequently, treatment-related adverse events and their impact on function and long-term quality of life are important in the context of cancer survivorship. Vincristine-induced peripheral neuropathy (VIPN) is a common and significant adverse event of vincristine treatment. VIPN causes sensorimotor peripheral neuropathy, presenting as numbness, tingling, weakness and pain in the distal extremities, as well as autonomic symptoms, including hypertension and bowel or bladder disturbances [2].

VIPN is highly pervasive among the paediatric cohort, affecting up to 78–100% of children treated with vincristine [3]. Motor symptoms are typically more severe than sensory [4], with potential to cause significant functional deficits [5]. Fortunately, the majority of children experience symptom improvement post vincristine treatment [6], although nerve dysfunction can be long-lasting [7].

The natural history of development and recovery of VIPN in the adult population, however, remains ill-described. Phase 3 clinical trials have suggested that VIPN may affect up 30–50% of adults treated with vincristine [8, 9]; however, these trials assessed VIPN using clinician-reported outcome measures such as the NCI-CTCAE, which underreport the incidence and severity of peripheral neuropathy [10]. Limited prior neurophysiological studies have demonstrated that VIPN may be sensory, as opposed to motor predominant in adults [11]. However, due to the lack of gold standard in VIPN assessment, an understanding of VIPN natural history and its phenotypic characteristics remain ill defined.

This study aimed to describe the natural history of VIPN in adults using a comprehensive battery of assessments including clinical grading, patient reported measures and semi-quantitative functional measures. Two study arms were employed, prospective and cross-sectional, in order to investigate the development of VIPN, as well as long-term outcomes.

## Methods

### Study design

Adult patients (aged > 18 years) prescribed vincristine therapy for the treatment of haematological cancers were recruited into an observational study of neuropathy outcomes from hospitals in Sydney, Australia [12]. Patients were eligible if were either about to commence vincristine treatment (prospective arm) or had completed treatment between 3 months and 5 years prior (cross-sectional arm). The study was approved by the Human Research Ethics Committees of South-Eastern Sydney and Sydney Local Health Districts and was conducted in accordance with the Declaration of Helsinki. All patients provided informed written consent prior to study participation.

**Fig. 1:**
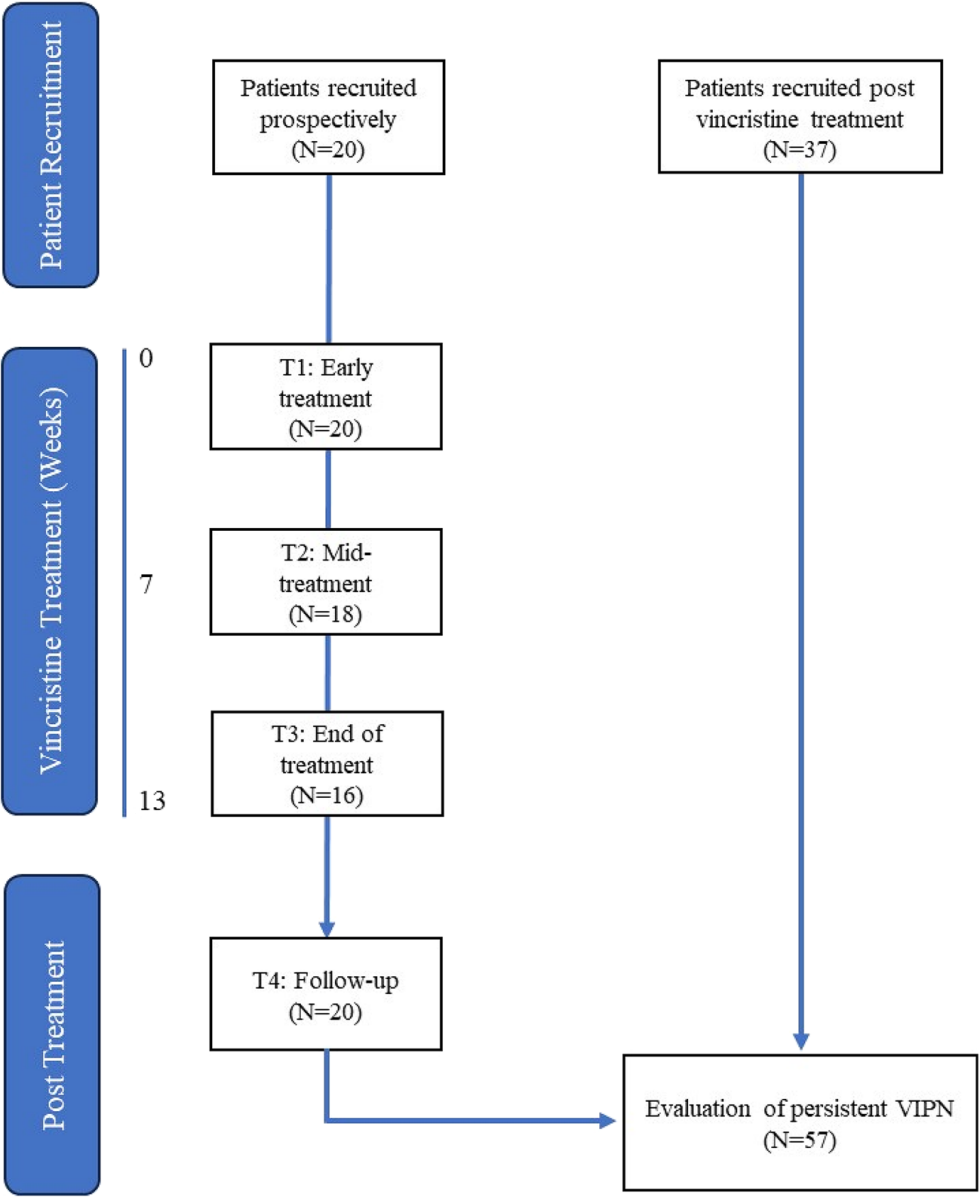
Study cohort and assessment timepoints

Patients were eligible for inclusion in this analysis if they had received a cumulative vincristine dose of ≥ 2.8 mg/m^2^. To phenotype persistent neuropathy, 57 patients who had completed vincristine treatment (median = 10 months, range = 3–56 months) were included for cross-sectional analysis. To examine the development and progression of VIPN, a subset of 20 patients who had been longitudinally evaluated for neuropathy outcomes were included for prospective analysis (Fig. 1). These patients were assessed at four timepoints: T1—Early treatment (*N* = 2 prior to vincristine treatment, *N* = 18 following single vincristine dose), T2—midway through prescribed treatment protocol, T3—upon completion of treatment protocol and T4—3–6 months after vincristine treatment.

### Assessment protocol

At each timepoint, patients completed a comprehensive battery of neuropathy assessments as described below, undertaken by trained researchers. All researchers received uniform neurologist-led training. Procedure protocols and training manuals were devised by senior researchers specifically for this study and were followed in all study visits. Assessors regularly met and worked together to ensure competence and quality control. Patient demographic (including age, BMI, diabetic status, cancer type) and neurotoxic treatment information (including dose, treatment frequency) was collected from medical records.

#### Patient reported outcome measures (PROMs)

The European Organization for Research and Treatment of Cancer Quality of Life Chemotherapy-Induced Peripheral Neuropathy Questionnaire (EORTC-QLQ-CIPN20) is a widely used validated measure of chemotherapy-induced peripheral neuropathy [13]. This 20-item questionnaire assesses the degree to which patients experienced each symptom on a 4-point Likert scale (1 = not at all, 4 = very much) [14]. Total scores were linearly transformed to a 0 to 100 scale, with higher scores representing greater symptoms. Factor analyses have not confirmed a subscale structure for the CIPN20 [15], and consequently, for the purpose of this analysis, questions were grouped according to symptom category. Questions assessing numbness and tingling were grouped as sensory symptoms (Q1–4), questions assessing muscle cramping and weakness as motor symptoms (Q7–8, 13, 15), questions assessing shooting or burning pain as pain symptoms (Q5–6) and questions assessing dizziness and blurry vision as autonomic symptoms (Q16–17). Questions were also grouped to investigate VIPN in the upper (Q1, 3, 5, 7, 13) or lower (Q2, 4, 6, 8, 15) limbs (Supplementary Table 1). All scores were also transformed to a 0–100 scale.

The Rasch Built Overall Disability Scale for patients with chemotherapy-induced peripheral neuropathy (CIPN R-ODS) is a PROM developed to evaluate disability in patients with chemotherapy-induced peripheral neuropathy [16]. This outcome measure consists of 28 items in which patients respond on their ability to complete each task (0—impossible to perform, 1—possible with some difficulty, 2—possible without any difficulty). Results are transformed to a linear scale ranging 0–100 with lower scores indicating greater disability.

#### Neurologically assessed CIPN

The Total Neuropathy Score—clinical version (TNSc © Johns Hopkins University) is a validated composite neurological grading scale designed to evaluate peripheral neuropathy [17, 18]. This measure consists of two patient reported items assessing the extent of sensory and motor symptoms as well as four items assessing quantifiable neuropathic signs (pinprick, vibration sensation, strength and deep tendon reflexes). Each item is scored 0–4 for a total score range of 0–24, with higher scores indicating greater neuropathy.

Nerve conduction studies (NCS) were completed in the left side of the lower limbs using a Nicolet EDX Synergy device (Natus Medical, Inc., Pleasanton, CA). NCS consisted of antidromic sural sensory nerve action potentials (SNAPs), recorded at the lateral malleolus with the stimulation site 10–15 cm proximal and tibial nerve compound muscle action potentials (CMAPs) recorded from the abductor hallucis muscle, stimulating posterior to the medial malleolus.

#### Semi-objective measures of sensation

The Grating Orientation Task (GOT) assessed tactile sensation on the fingertips using JVP Domes (Stoelting Co) with dome gratings ranging between 0.75 and 12 mm wide. Domes were pressed onto the index finger of the dominant hand either proximal-distally or lateral-medially in random order to identify the smallest grating that could be reliably discriminated [19]. The Two-Point Discriminator Task (2PD) assessed spatial sensation in distal lower limbs. An aesthesiometer was placed on the first toe, and participants were required to correctly differentiate between one and two points (between 2 and 15 mm) 7 out of 10 times [20]. Fine motor skills were assessed using the grooved pegboard task. Time taken for participants to place 25 pegs with the dominant hand into grooved holes were recorded, with the final score being an average of two attempts [21].

### Statistical analysis

Summary statistics are presented as mean and standard deviation or median and interquartile range (IQR). The presence or absence of VIPN was categorised by whether patients reported core VIPN symptoms on the CIPN20: sensory (Q1, 2, 3, 4), motor (Q7, 8, 13, 15) or painful (Q5, 6), (Supplementary table). Comparisons between outcome measures are calculated with independent sample *t* tests or Mann–Whitney *U* tests for non-parametric data. Comparison of data between timepoints was calculated using paired sample *t* tests or Wilcoxon matched-pairs signed-rank test. Results are presented as mean ± standard error or median (IQR). Correlations are presented as Spearman’s rho, with 0.3, 0.5 and 0.8 indicating weak, moderate and strong correlations [22]. Comparisons between two observed groups are calculated using chi-square. Clinical risk factors (age, sex, body mass index (BMI), diabetic status and cumulative vincristine dose (mg/m^2^)) associated with persisting VIPN was investigated with univariate logistic regressions presented and as odds ratios (OR). Statistical significance was defined at *P* < 0.05. All statistical analyses were performed using Stata version 14 (StataCorp, College Station, TX, USA).

## Results

Fifty-seven patients were included in the analysis (Table 1; Fig. 1). Patients were predominantly treated for Non-Hodgkin’s (82.5%, *n* = 47) or Hodgkin’s lymphoma (7.0%, *n* = 4) and received a mean cumulative dose of 7.6 ± 4.4 mg/m^2^ of vincristine (range 2.8–11.2 mg/m^2^). There were no differences in demographic (age, sex) or dosing factors between patients recruited prior to (*n* = 20) or post (*n* = 37) vincristine treatment (Supplementary Table 2).

**Table 1:** Patient demographic information

	Cross-sectional *N* = 57	Prospective *N* = 20
Sex (%)		
Male	39 (68.4%)	12 (60.0%)
Female	18 (31.6%)	8 (40.0%)
Age (years)		
Mean (SD)	59.8 (14.6)	55.1 (15.8)
BMI (SD)	27.9 (5.2)	27.6 (3.3)
Diabetes (%)		
Yes	7 (12.3%)	1 (5.0%)
Cancer type (%)		
Non-Hodgkin’s Lymphoma	47 (82.5%)	16 (80.0%)
Hodgkin’s lymphoma	4 (7.0%)	2 (10.0%)
Brain	2 (3.5%)	2 (10.0%)
B-cell leukaemia	2 (3.5%)	-
Prostate	1 (1.8%)	-
Primitive neural Ectodermal tumour	1 (1.8%)	-
Cumulative vincristine dose (mg/m^2^)		
Mean (SD)	7.6 (2.2)	7.6 (2.1)
Vincristine frequency		
2-weekly	22 (38.6%)	9 (45%)
3-weekly	33 (57.9%)	11 (55%)
6-weekly	2 (3.6%)	-
Time since treatment completion (months)		
Mean (SD)	16.3 (15.6)	7.6 (5.4)

### Cross-sectional analysis: persistent VIPN

At 16.3 ± 15.6 months following vincristine treatment, 80.7% (*n* = 46/57) of patients reported persistent VIPN (EORTC-QLQ-CIPN20). To account for the range of time since vincristine completion, patients were categorised by time since treatment (less than 1 year (*n* = 29), 1 to 2 years (*n* = 15) and greater than 2 years (*n* = 13)). Comparison of neuropathy severity assessed via the CIPN20 found no significant difference between the three cohorts (*P* > 0.05).

#### Patient reported VIPN presentation

**Fig. 2:**
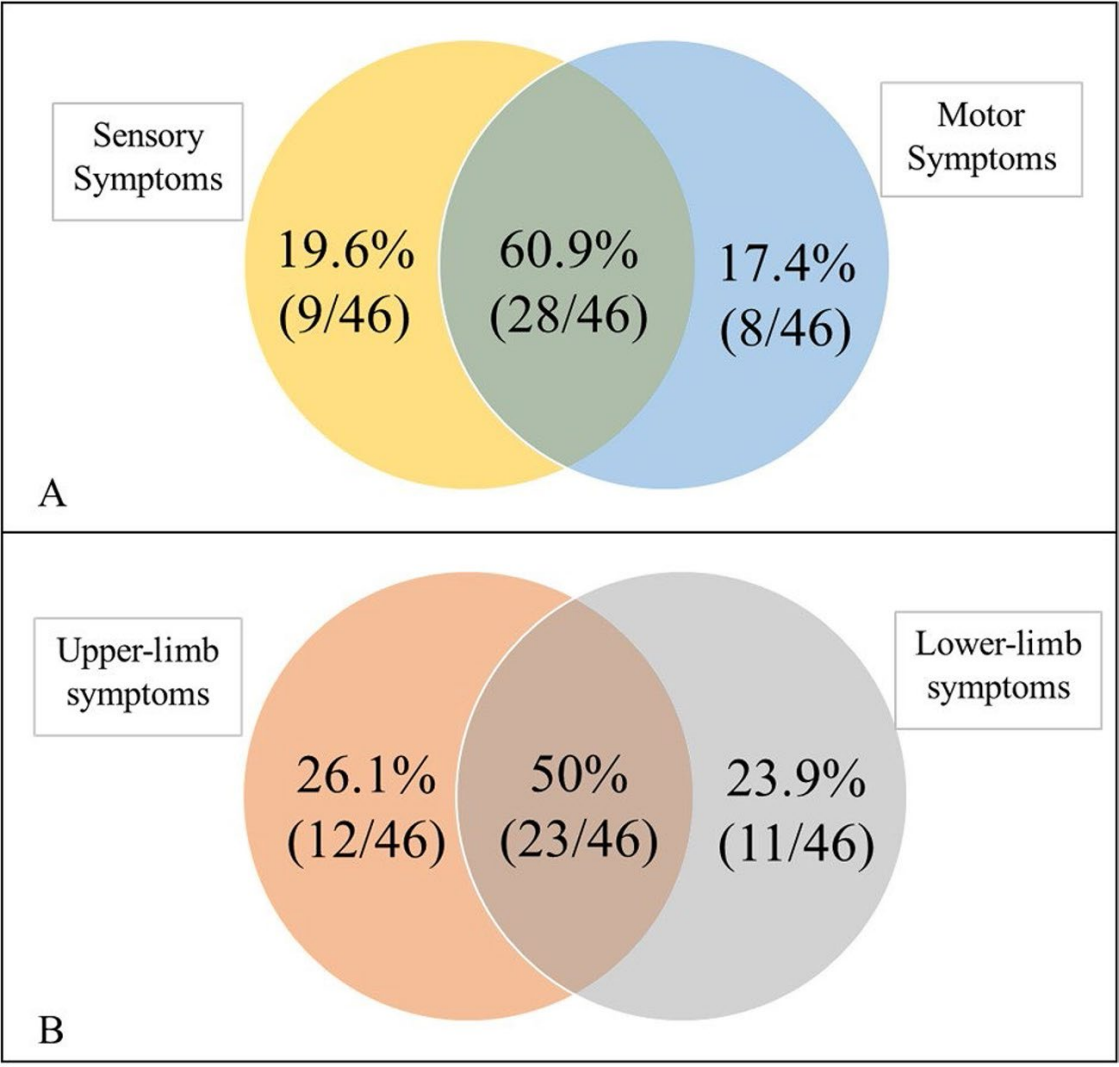
Persistent VIPN presentation by **A** sensory and motor symptoms and **B** upper- and lower-limb symptoms

Among those with persistent VIPN (*n* = 46), similar proportions reported sensory (80.4%, 37/46) or motor (78.3%, 36/46; *P* > 0.05) symptoms of neuropathy (EORTC-QLQ-CIPN20). The majority of patients reported both sensory and motor symptoms (60.9%, 28/46) (Fig. 2A), with no difference in reported symptom severity between sensory or motor symptom scores (*P* > 0.05). Autonomic dysfunction including dizziness or blurry vision was reported in 29.8% (*n* = 17/57) of patients. Reports of upper limb (76.1%, 35/46) symptoms were also proportionally similar to lower limb (73.1%, 34/46; *P* > 0.05), with 50% (23/46) of patients reporting symptoms in both upper and lower limbs (Fig. 2B). However, symptoms in the lower limb were more severe than in the upper limbs (20.6 ± 3.1 vs 12.9 ± 1.8 m *P* < 0.01). Shooting and burning pain was not a common feature, presenting in 26.1% (12/46) of patients with VIPN. The severity of persistent VIPN was significant (described as “quite a bit” or “very much” in at least one VIPN item on the CIPN20; Supplementary table) in 58.7% of patients (27/46).

#### Impact of CIPN on neurological function and disability

Neurologically graded CIPN (TNSc) was moderately correlated with patient-reported CIPN (CIPN20, *r* = 0.38, *P* < 0.01). Overall disability (R-ODS) was strongly correlated with patient-reported CIPN (CIPN20, *r* =  − 0.72, *P* < 0.01), indicating patients with greater symptoms reported increased overall disability.

Patients reporting VIPN (*n* = 46) had significantly higher TNSc scores than patients who did not report VIPN symptoms (*n* = 11) (4(3.5) vs 1(4), *P* < 0.05). Patients with VIPN took longer to complete the grooved pegboard task (73.7(27.6) vs 65.9(17.05) s, *P* < 0.01) and had larger sensory thresholds in upper and lower limbs or sensory acuity (GOT 3.7 ± 0.2 vs 2.6 ± 0.1 mm; 2PD 11.6 ± 0.5 vs 8.5 ± 0.6 mm; both *P* < 0.01) than patients who did not report VIPN.

Patients reporting VIPN had smaller sural SNAPs (7.0(6.2)µV vs 18.1(11.9)µV) and tibial CMAPs (7.8 ± 0.6 mV vs 13.0 ± 1.3 mV) than patients who did not report VIPN, and this was significant even accounting for age (both *P* < 0.05).

#### Clinical factors associated with VIPN

On univariate analysis, age was the only baseline clinical risk factor associated with the presence of patient reported VIPN. Each year increase in age was associated with a 9% increased risk of persistent VIPN (OR = 1.09, *P* < 0.05). There were no increased odds associated with cumulative vincristine dose, diabetic status, sex or BMI (all *P* > 0.05).

### Prospective analysis—development and recovery of VIPN

Twenty participants were prospectively evaluated to investigate the development and recovery of VIPN (Fig. 1).

**Fig. 3:**
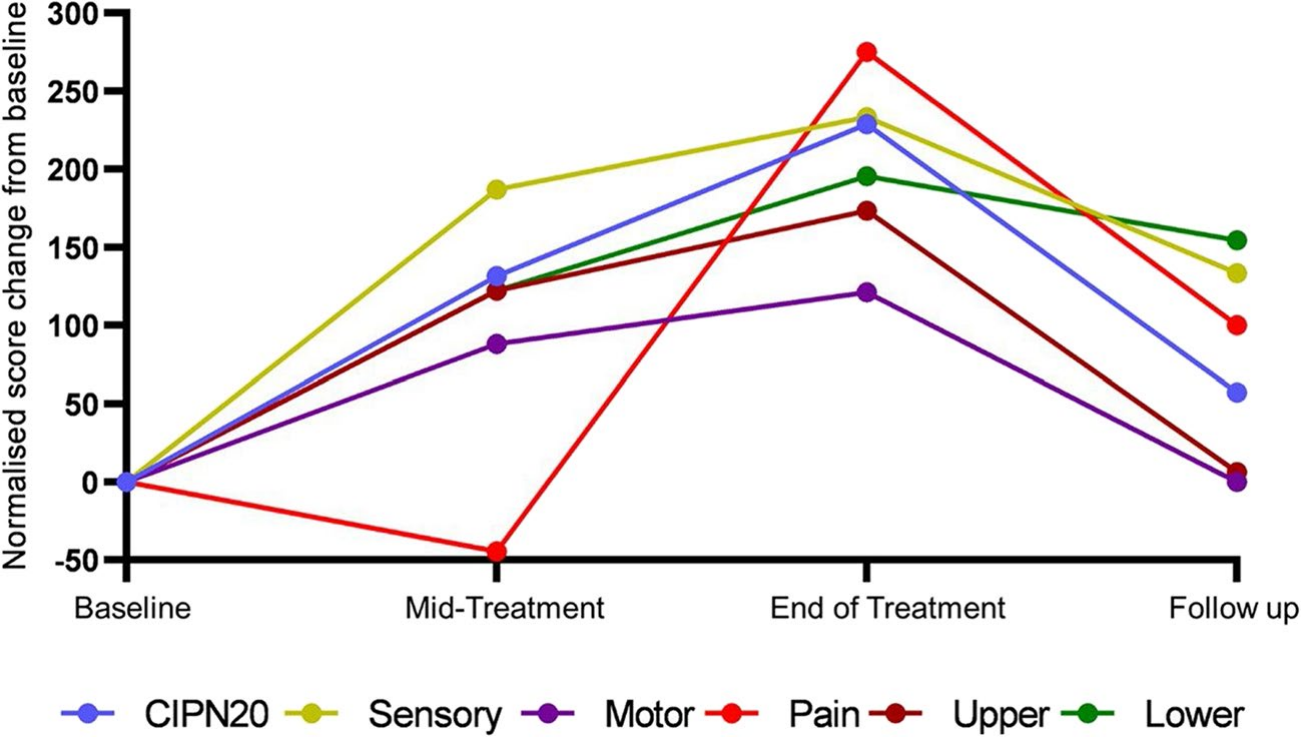
Time course of VIPN symptoms as assessed using EORTC-CIPN20 subscales, normalised to baseline scores

At T2 (7.0 ± 3.3 weeks post T1), 77.8% of patients reported VIPN (14/18) (Supplementary Table 1). Overall CIPN20 scores significantly worsened compared to T1 (Table 2). In particular, patient-reported sensory, motor and lower limb symptoms demonstrated significant worsening (Table 2, Fig. 3). There were similar proportions of patients with VIPN reporting motor (78.6%, 11/14) and sensory (71.4%, 10/14; *P* > 0.05) symptoms although majority of patients reported both sensory and motor involvement (57.1%, 8/14). Only one patient reported painful VIPN. Prevalence of symptoms in the hands (95.6%, 13/14) and feet (85.7%, 12/14) were similar, with most patients (78.6%, 11/14) reporting both upper and lower limb involvement. Autonomic symptoms were reported in 38.9% (7/18) of patients, compared to 15.0% (3/20) at T1; however, this difference was not significant (*P* > 0.05).

**Table 2:** Difference in assessments scores between timepoints presented as mean (SE)

Assessment	Baseline to mid-Treatment T1 – T2	Mid-treatment to end-of-treatment T2 – T3	End-of-treatment to baseline T1 – T3	Baseline to follow-up T1 – T4
Patient reported symptoms				
CIPN20	4.65 (1.79)*	1.76 (2.04)	7.81 (2.31)*	1.89 (1.70)
Sensory subscale	8.80 (4.74)*	0.00 (0.91)	13.54 (4.43)*	6.67 (4.34)
Motor subscale	6.02 (2.68)*	1.79 (1.89)	7.29 (3.03)*	0.00 (2.90)
Pain subscale	0.00 (0.35)	1.19 (1.19)	4.17 (4.44)	1.67 (2.39)
Upper limb subscale	5.93 (3.88)	0.95 (3.34)	11.25 (4.20)*	0.33 (2.92)
Lower limb subscale	5.93 (2.08)*	0.00 (1.98)	7.08 (2.31)*	5.67 (3.22)*
Neurological evaluation				
TNSc	1.18 (0.54)*	1.4 (0.58)*	3.33 (0.92)*	1.40 (0.7)*
Sural SNAP (µV)	1.73 (1.31)	1.75 (0.19)	2.89 (0.13)*	0.59 (0.94)
Tibial CMAP (mV)	0.14 (0.63)	1.48 (0.95)	1.62 (0.88)*	1.44 (0.69)*
Functional/sensory assessment				
2-point Discrimination (mm)	1.17 (0.64)*	0.63 (0.73)	1.63 (0.86)	0.67 (0.66)
Grating orientation (mm)	0.65 (0.20)*	0.11 (0.32)	0.22 (0.24)	0.05 (0.24)
Grooved pegboard (mN)	2.46 (1.67)	4.53 (0.155)*	7.08 (2.22)*	2.18 (1.38)

Sample size for each timepoint are as follows: T1 = 20, T2 = 18, T3 = 16, T4 = 20

^*^Denotes significant score change at *P* < 0.05

By T3 (8.1 ± 1.7 weeks post T2), CIPN severity remained stable, with no significant worsening in CIPN20 scores compared to T2 (Table 2) and a similar proportion of symptomatic patients (75%, 12/16; *P* > 0.05) reporting VIPN. However, severity for all patient-reported symptom categories except for pain were significantly greater than T1 (Fig. 3). Sensory and motor symptoms were equally prevalent (both 75.0%, 9/12) with 50.0% (6/12) of patients with VIPN reporting both sensory and motor symptoms. The majority of patients (75.0%, 9/12) reported both upper and lower limb involvement.

At T4, half of patients (50%, 10/20) reported persistent VIPN. Overall CIPN20 as well as subscale scores had improved compared to T3 and were no longer different to T1, except for lower limb symptoms (Table 2). In the patients with persisting symptoms (*n* = 10), 90% (9/10) reported sensory and 60% (6/10) reported motor symptoms; however, this difference was not significant (*P* > 0.05). Rates of autonomic symptoms also dropped to baseline levels, at 10.0% (2/20).

#### Neurological evaluation of VIPN

On neurological assessment, TNSc scores significantly worsened by T2 compared to T1 (Table 2). However, reduced sensation was not commonly observed in patients with VIPN at T2 (reduced pin-prick or vibration sense in 14.3%, 2/14). No patients presented abnormal strength, and 50.0% (7/14) of patients who reported VIPN had reduced reflexes at T2. Nerve conduction studies established no significant change in either sural or tibial nerve amplitudes (Table 2).

**Fig. 4:**
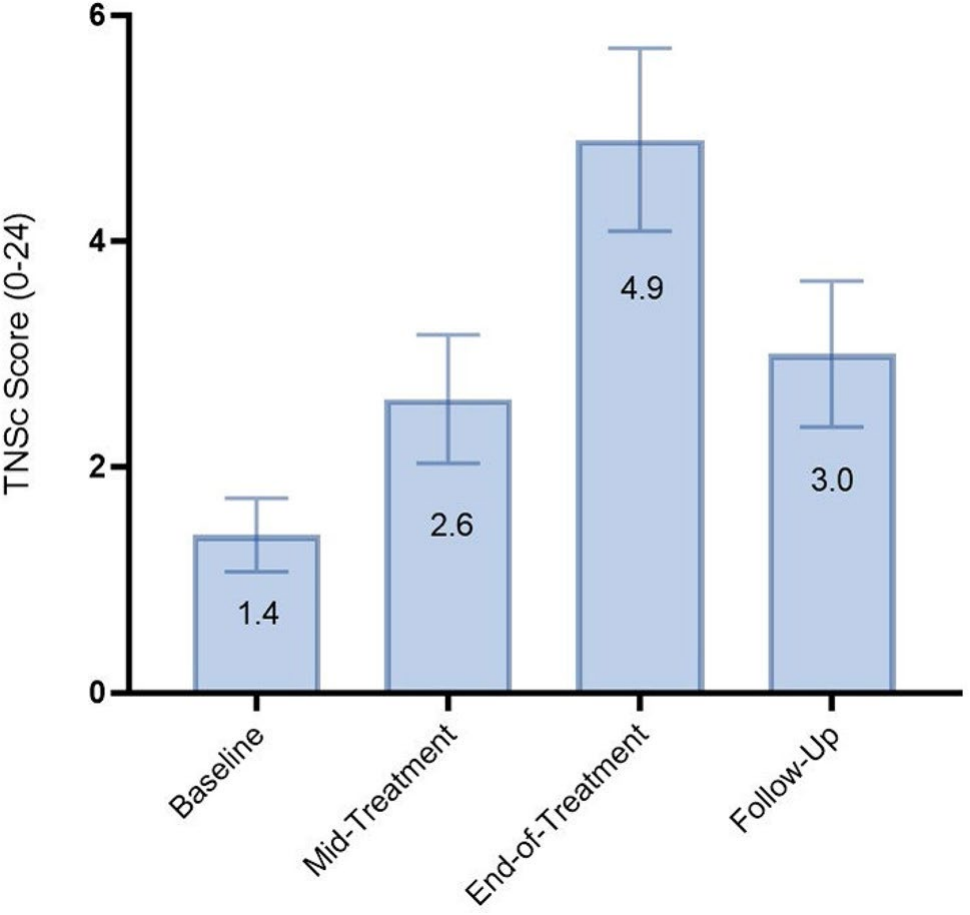
Mean TNSc scores at each timepoint. Error bars indicate standard errors

TNSc scores continued to decline from T2 to T3 (Table 2) where it reached nadir (Fig. 4). In patients with VIPN, 44.4% (4/9) had reduced pin-prick, 40% (4/10) reduced vibration sensation, 20% (2/10) reduced strength and 70% (7/10) reduced reflexes. Mean sural and tibial NCS amplitudes did not differ significantly from T2. However, compared to T1, TNSc scores, sural and tibial amplitudes had significantly decreased (Table 2).

By T4 neurological grading demonstrated improvement compared to T3 (TNSc score diff =  − 2.5 ± 0.01, *P* < 0.05) but was still significantly worse compared to T1 (Table 2). Sural amplitudes returned to baseline values; however, tibial amplitudes remained reduced (Table 2).

#### Functional assessment of VIPN

Sensory perception in the fingers and toes declined by T2 with no further decline by T3 (Table 2). Fine motor function deficits were only evident by T3 compared to T1, with significant decline on the grooved pegboard task. Overall, at T4, all functional assessments had returned to T1 levels. However, in patients reporting persisting symptoms, fine motor function was still reduced compared to T1 (Pegboard diff = 2.59 ± 1.26 s, *P* < 0.05).

## Discussion

To address a gap in current understanding about chemotherapy-induced peripheral neuropathy, the present study has provided an updated natural history analysis of VIPN from baseline to 3 months post treatment, with an examination of chronic VIPN. Compared to prior investigations of VIPN undertaken more than 20 years ago [11, 23, 24], this study evaluated VIPN using current dosing regimens. Recent studies have utilised retrospective chart review or NCI-CTCAE grading to define neuropathy prevalence and characteristics [25–27]. However, this clinical study utilised a comprehensive and multifaceted assessment approach, incorporating patient reported, neurological and functional perspectives.

Vincristine produces a chronic sensorimotor dysfunction associated with functional impact. Most patients reported VIPN development by mid-treatment, without further significant change by end-of-treatment. Post treatment recovery occurred within 3 months in a proportion of patients and was associated with improved scores on neurological grading and functional assessment.

### Impact of VIPN

While the present study determined that 78% of patients developed VIPN, previous studies have suggested a large range of reported prevalence, from 44 to 92%, across studies [24, 28]. This most likely reflects the range of methods utilised for assessing neuropathy, with the most commonly used measure in large-scale clinical trials, NCI-CTCAE, known to have low inter-rater reliability [10]. Accordingly in the present study, VIPN assessment included patient symptom report as patient reported measures are becoming increasingly recognised as a valuable tool to capture neuropathy information [29]. Another important consideration that may affect reported VIPN prevalence is that the NCI-CTCAE sensory neuropathy subscale is most commonly used in clinical trials, which does not incorporate motor neuropathy symptoms, which is also a critically important characteristic of VIPN.

At mid-treatment, VIPN symptoms had reached the threshold of clinical significance (score increase of > 4.26 on the CIPN20) [30]. VIPN presented as motor and sensory neuropathy, affecting upper and lower limbs. Although prior studies investigating VIPN phenotypes are limited, early studies suggested VIPN to be predominantly sensory [23, 31], while others have demonstrated motor involvement is also significant on clinical [11, 32] and electrophysiological [33, 34] examination. This significant motor neuropathy development is characteristic of vincristine neurotoxicity. Other neurotoxic agents including taxanes, platinums, bortezomib and thalidomide are typically associated with only minor motor neuropathic symptoms [35]. Autonomic symptoms were more common in this current cohort compared to prior studies (30–39% versus 19%, [25]), and this is likely due to the varying methods of assessing autonomic neuropathy. While the present study utilised the autonomic items of the EORTC-QLQ-CIPN20, prior studies have highlighted limitations to this autonomic subscale [15]. Future studies should assess autonomic symptoms in vincristine-treated patients more directly to investigate the burden of autonomic nerve damage following vincristine.

The ‘coasting’ phenomenon, whereby symptoms worsen after end of treatment, was also previously associated with VIPN [24]. However, the present study incorporating multiple assessment techniques found that symptoms had reached their peak by the end of treatment. As observed in a prior study [36], symptom improvement occurred soon after treatment completion with significant improvements in 3 months post vincristine. This discrepancy may be due to dosing differences, as current practice is to cap each vincristine infusion at 2 mg, and the dosing limit was not applied in the earlier study where 90% of patients had a first dose > 2 mg [24]. In patients that experience improvement, the timecourse of VIPN recovery is similar to that seen in paclitaxel- and bortezomib-induced peripheral neuropathy, where symptom improvement occurs by 3 months after treatment completion [37, 38]. However, this pattern of recoverability is different to platinum agents, where symptoms may worsen for up to 6 months post treatment [39].

Despite the resolution of VIPN in a proportion of patients, this analysis of chronic VIPN identified significant symptoms which lasted more than 1 year post treatment. Persistent VIPN was associated with reduced spatial acuity in both the upper and lower limbs, reduced fine motor function, reduced sensory and motor nerve amplitudes and increased overall disability. This profile of long-term neuropathy symptoms is similar to deficits resulting from peripheral neuropathy due to other chemotherapies including taxanes and platinums [12, 40].

### Adult and paediatric VIPN

In the paediatric cohort, VIPN typically presents as motor predominant [4, 41, 42]. However, sensory neuropathy is difficulty to evaluate in young children, as they may have difficulty in communicating their experiences of sensory symptoms such as ‘numbness and tingling’. On the other hand, motor neuropathy is easier to clinically detect, with patients often being visibly weaker, and less active. Conversely in adults, ‘numbness and tingling’ is a unique abnormal sensation that patients can often easily recognise and report, whereas motor neuropathy symptoms including weakness and cramping are often difficult to attribute solely to treatment side-effects as opposed to cancer or age-related deconditioning.

Electrophysiological examinations in paediatric VIPN have also demonstrated greater motor compared to sensory nerve involvement [4, 43, 44] suggesting the motor-predominant neuropathy may not purely be due to symptom communication difficulties, or due to myopathy. While specific reasons behind this difference remains unknown, the difference in phenotypes may be related to age-dependent molecular changes in axonal function which may affect repair or regeneration pathways [45, 46]. Experimental models of traumatic injury or inherited neuropathy have also demonstrated differences in phenotypic expression of symptoms based on age [47, 48], highlighting that age-dependent changes in axonal function may influence VIPN presentation between adults and children. In the present adult study, there were reductions in both motor and sensory amplitudes by end of treatment; however, patients reporting persisting symptoms did not have lower amplitudes than patients without neuropathy. This may be related to inter-individual variability in baseline amplitudes. Further, electrophysiological, clinical and patient reported approaches to neuropathy assessment have been demonstrated to not directly conform [49, 50], suggesting each outcome measure alone is not sufficient to entirely capture the severity, impact and experience of neuropathy. Discrepancies between clinical examination and neurophysiological studies are common [50] and potentially reflect the different sensitivity of techniques to identify dysfunction.

### Limitations

This study was an observational natural history investigation of VIPN, with a limited sample size. Due to the nature of longitudinal research, not all prospective patients were able to attend all testing timepoints, and the authors acknowledge this as a limitation to the study. Future studies should conduct a priori power analyses to determine the sample sizes needed to detect change in neuropathy signs and symptoms. This will also reduce chances Type 1 errors, which may have occurred in this present study. Furthermore, longer-term follow-ups may provide greater insight in VIPN recovery as well as patterns of adaptation in chronic VIPN. NCS were only performed on the lower limbs in the present study, and future studies should also investigate neurophysiological changes in the upper limbs. Assessment of autonomic neuropathy was brief in this study, and future studies should incorporate more rigorous outcome measures, including objective markers of autonomic neuropathy to detail the severity and impact of autonomic neuropathy. As some patients were recruited cross-sectionally, the prevalence of VIPN in this cohort needs to be considered in this context as it may be higher than a purely prospective study. Cross-sectional participants were also recruited from a range of time since vincristine treatment, and vincristine regimens. While this was intentional in order to examine a representative sample of chronic VIPN presentation, this has resulted in a heterogenous participant sample. Other chemotherapies taken concurrently with vincristine were not controlled for; however, it is unlikely that this would have affected the profile of VIPN. In order to more fully identify risk factors for VIPN development such as cumulative dose, larger prospective studies are necessary. A number of genetic polymorphisms have been associated with VIPN, particularly in children [51]. These should be interrogated in prospective series utilising comprehensive neuropathy assessment protocols in order to better guide identification of patients at risk of long-term sequelae.

## Conclusions

The present study underscores the importance of examining neuropathy symptom profiles within different age groups and clinical cohorts, using a multifaceted approach. The results provide a natural history of VIPN from development to recovery, with a further snapshot of chronic persistence post treatment completion. Understanding the clinical presentation of VIPN and the recovery profile, as well as the functional impact of neuropathy, will enable rehabilitation and management strategies to be targeted to patients with persistent functional disability most in need of intervention.

### Supplementary Information

Below is the link to the electronic supplementary material.

Supplementary file1 (PDF 307 KB)

## Data Availability

Datasets are available from the corresponding author upon reasonable request.
