# Supplementary material for: Characterising vincristine-induced peripheral neuropathy in adults: symptom development and long-term persistent outcomes
Source: Support Care Cancer. 2024 Apr 9;32(5):278. doi: 10.1007/s00520-024-08484-5 (PMC11003903; doi:10.1007/s00520-024-08484-5)
Supplement: Supplementary file 1 — (PDF 307 KB) [file 520_2024_8484_MOESM1_ESM.pdf]

Supplementary

Table 1. Items of EORTC CIPN20 used to form subgroups for this analysis.

	**VIPN**	**Sensory**	**Motor**	**Pain**	**Upper limbs**	**Lower limbs**	**Autonomic**
Q1. Did you have tingling fingers or hands?	●	●			●		
Q2. Did you have tingling toes or feet?	●	●				●	
Q3. Did you have numbness in your fingers or hands?	●	●			●		
Q4. Did you have numbness in your toes or feet?	●	●				●	
Q5. Did you have shooting or burning pain in your fingers or hands?	●			●	●		
Q6. Did you have shooting or burning pain in your toes or feet?	●			●		●	
Q7. Did you have cramp in your hands?	●		●		●		
Q8. Did you have cramp in your feet?	●		●			●	
Q9. Did you have problems standing or walking because of difficulty feeling the ground under your feet?							
Q10. Did you have difficulty distinguishing between hot and cold water?							
Q11. Did you have a problem holding a pen, which made writing difficult?							
Q12. Did you have difficulty manipulating small objects with your fingers (for example, fastening small buttons)?							
Q13. Did you have difficulty opening a jar or bottle because of weakness in your hands?	●		●		●		
Q14. Did you have difficulty walking because your feet dropped downwards?							
Q15. Did you have difficulty climbing stairs or getting up out of a chair because of weakness in your legs?	●		●			●	
Q16. Were you dizzy when standing up from a sitting or lying position?							●
Q17. Did you have blurred vision?							●
Q18. Did you have difficulty hearing?							
Q19. Did you have difficulty using the pedals?							
Q20. Did you have difficulty getting or maintaining an erection?							

Table 2. Comparison between prospective and cross-sectional patients included in persistent VIPN analyses.

	Prospective (n=20)	Cross-Sectional (n=37)	P value
Age (Mean ± SE)	56.0 ± 3.6	61.9 ± 2.2	>0.05 (Mann-Whitney U)
Sex (F:M %)	40%:60%	27%:73%	>0.05 (Chi-square)
Cumulative vincristine dose (mg/m^2) (Mean ± SE)	7.7 ± 0.3	7.6 ±0.4	>0.05 (T-test)
